# Who needs collaborative care treatment? A qualitative study exploring attitudes towards and experiences with mental healthcare among general practitioners and care managers

**DOI:** 10.1186/s12875-018-0764-z

**Published:** 2018-05-30

**Authors:** Marlene Christina Rosengaard Møller, Anna Mygind, Flemming Bro

**Affiliations:** Research Unit for General Practice & Section for General Medical Practice, Department of Public Health, Aarhus University, Bartholins Allé 2, 8000 Aarhus C, Denmark

## Abstract

**Background:**

Collaborative care treatment is widely recognized as an effective approach to improve the quality of mental healthcare through enhanced and structured collaboration between general practice and specialized psychiatry. However, studies indicate that the complexity of collaborative care treatment interventions challenge the implementation in real-life general practice settings. Four Danish Collaborative Care Models were launched in 2014 for patients with mild/moderate anxiety and depression. These involved collaboration between general practitioners, care managers and consultant psychiatrists.

Taking a multi-practice bottom-up approach, this paper aims to explore the perceived barriers and enablers related to collaborative care for patients with mental health problems and to investigate the actual experiences with a Danish collaborative care model in a single-case study in order to identify enablers and barriers for successful implementation.

**Methods:**

Combining interviews and observations of usual treatment practices, we conducted a multi-practice study among general practitioners who were not involved in the Danish collaborative care models to explore their perspectives on existing mental health treatment and to investigate (from a bottom-up approach) their perceptions of and need for collaborative care in mental health treatment. Additionally, by combining observations and qualitative interviews, we followed the implementation of a Danish collaborative care model in a single-case study to convey identified barriers and enablers of the collaborative care model.

**Results:**

Experienced and perceived enablers of the Danish collaborative care model mainly consisted of a need for new treatment options to deal with mild/moderate anxiety and depression. The model was considered to meet the need for a free fast track to high-quality treatment. Experienced barriers included: poor adaptation of the model to the working conditions and needs in daily general practice, time consumption, unsustainable logistical set-up and unclear care manager role. General practitioners in the multi-practice study considered access to treatment and not collaboration with specialised psychiatry to be essential for this group of patients.

**Conclusions:**

The study calls for increased attention to implementation processes and better adaptation of collaborative care models to the clinical reality of general practice. Future interventions should address the treatment needs of specific patient populations and should involve relevant stakeholders in the design and implementation processes.

**Electronic supplementary material:**

The online version of this article (10.1186/s12875-018-0764-z) contains supplementary material, which is available to authorized users.

## Background

Collaborative care is a widely recognized approach to improve mental healthcare through enhanced and systematic collaboration between general practice and specialized psychiatry. Trials have shown better outcomes with collaborative care for patients with mental health problems when compared to usual care [1–10]. However, trial evidence seems difficult to translate into real-life care delivery [8, 11–17]. Danish Collaborative Care Models (DCCMs) for patients with mild/moderate depression and anxiety were launched in four (of five) Danish regions in 2014. The intervention designs of these DCCMs were in line with the recommendations in existing literature on collaborative care [18–21]. Gask et al. [22] defines CC treatment by four key components: (1) a multi-professional approach to patient care, (2) a structured management plan in the form of guidelines or protocols for interventions, e.g. antidepressant medication, patient screening, education, counselling, cognitive behavior therapy, (3) scheduled patient follow-up, and (4) enhanced inter-professional communication, e.g. team meetings, case conferences, individual consultation/supervision, shared medical records, patient-specific written or verbal feedback between caregivers.

The model explored in this study involved collaboration between general practitioner (GP) and care managers (often psychiatrically trained nurses) from specialized psychiatric units in the Central Denmark Region. Care managers offered a 12-week treatment course to patients in general practice. The course consisted of 5–6 individual Cognitive Behavioural Therapy-based sessions; first and last sessions were shared with the patient’s GP. Other components were questionnaires for diagnosing and monitoring, group-based psycho-education, supervision and training of GPs, and weekly supervision of care managers by a psychiatrist. Each care manager collaborated with up to ten GPs.

The GPs were responsible for identifying eligible patients, arranging appointments with the care manager and the patient for a shared consultation and subsequently referring patients electronically through existing psychiatric visitation procedures by adding a reference to the specific care manager assigned the GP. Moreover, GPs held the treatment responsibility throughout the course of collaborative care treatment and provided a treatment room for the care manager. GPs were remunerated for the time spent on clinical conferences with the care managers.

The DCCM was designed by specialised psychiatry to engage GPs in a collaboration aiming to improve both the treatment of patients and the GPs’ skills in psychiatric treatment through intensified cooperation across scientific disciplines and sector boundaries [23].

### Aim

This paper aims to explore the experiences with current treatment practices among GPs, clinic staff and care managers and to examine their views on and perceptions of future collaborative care. The paper also aims to identify enablers and barriers for successful implementation of a specific DCCM. The study provides new knowledge on a sparsely researched topic by taking a qualitative approach to exploring experiences with and perceptions of collaborative care in a general practice setting.

## Methods

### Design and setting

We employed a qualitative approach and used a combined research design to conduct a multi-practice study among GPs together with a single-case study of the DCCM.

General practice clinics in Denmark are privately owned by GPs and organised in small units, either as single-handed practices (1 GP) or group practices (2–10 GPs) [24]. Most single-handed practices also have a medical receptionist and/or a nurse, whereas group practices usually have both types of clinic staff. More than 95% of the Danish population is listed with a specific GP (approximately 1600 patients per GP), and all inhabitants must obtain a referral from their GP to get specialist treatment. Treatment is tax-funded, and health services are divided into primary care (including general practice) and secondary care (including hospitals and specialised psychiatry) with separate organisations and different financial structures. Psychologists generally operate on a private basis, but GP-referred patients are entitled to partial reimbursement. Danish GPs treat patients with various degrees of mental health problems. For the larger patient population with mild/moderate anxiety and depression, usual care often consists of a limited number of conversational therapy consultations with the GP and in some cases medical treatment. Only in severe cases of mental health problems, GPs may refer patients to treatment in specialised psychiatry.

### Sampling and recruitment

We conducted a multi-practice study to explore the experiences of GPs and their staff with current mental health treatment and to examine their perceptions of and need for collaborative care. The practices were selected among general practices that did not take part in the DCCM. To ensure diversity of opinion and broad general practice representation, we applied a purposive sampling strategy employing three main parameters: urban/rural location, practice size and proximity to specialised psychiatry. We thus identified clinics from three different geographic areas of the Central Denmark Region. We included a group practice located in an urban area in close vicinity of specialised psychiatry, a single-handed practice located in a rural area in long distance from specialised psychiatry, and a large group practice located in another rural area a bit closer to specialised psychiatry. Some GPs were known for their involvement in quality development and continuing education, whereas others were not. We included 10 GPs in our observational studies. Nine GPs participated in semi-structured interviews (Table 1). None of the GPs were personally or professionally acquainted with the anthropologist performing observations and interviews.

**Table 1:** Study participants and their participation in research activities

Participants	Practice type	Semi-structured interview	Follow-up interview	Ethnographic conversation	Direct observation	Participation in DCCM
Single-case study						
GP 1, practice 1	Single-handed, urban	x	x	x	x	x
Care manager 1		x	x	x	x	x
Care manager 2		x		x		x
Multi-practice study						
GP 2, practice 2	Group, rural	x		x	x	
GP 3, practice 2	Group, rural	x		x	x	
GP 4, practice 2	Group, rural	x		x	x	
GP 5, practice 2	Group, rural	Cancelled		x	x	
GP 6, practice 2	Group, rural	Left job		x	x	
GP 7, practice 3	Solo, rural	x		x	x	
GP 8, practice 4	Group, urban	x		x	x	
GP 9, practice 4	Group, urban	x		x	x	
GP 10	Group, urban	x				
GP 11	Group, urban	x				
Total		9	2	11	10	3

Abbreviations: *GP* general practitioner, *DCCM* Danish Collaborative Care Model

For the single-case study, we recruited a GP among the 15–20 GPs attending the information meeting hosted by the DCCM project managers in one of the participating municipalities. The recruited GP runs a single-handed practice located in an urban area with the assistance of a receptionist and a medical intern.

The care manager affiliated with the GP in the single-case study also agreed to participate in the single-case study. The care manager also collaborated with other GPs participating in the DCCM. Her participation was cleared with the DCCM management. A second care manager was recruited at the DCCM information meeting in the other DCCM participating municipality. The experiences of the second care manager helped qualify the findings in the single-case study and provided valuable insights into the workings of the DCCM. Moreover, since care managers involved in the DCCM collaborated with several general practitioners, the care manager in the single-case study was able to relate the single-case study experiences with her experiences with the DCCM in general.

### Data collection

#### Observational studies

To explore current clinical practices and establish an understanding of the context of mental health treatment, we observed a number of GP-patient consultations and practice nurse-patient consultations in the multi-practice study and in the single case study before the launch of the DCCM. The first author (MM) spent 2–6 days in each included clinic to explore how GPs treat patients with various degrees of mental health problems. In the multi-practice study, we conducted a total of approximately 80 h of observations; these were evenly distributed between the GPs. Consequently, more hours of observations were done in clinics with more GPs.

To get insight into various DCCM components and to deepen our understanding of both the context and specific barriers and enablers, we followed one patient in the single-case study throughout the entire collaborative care treatment period through multiple observations and interviews with both GP and care manager. We conducted participant observations of their collaborative work through each step of the intervention, including their initial preparation meeting, the first shared consultation, the individual sessions between patient and care manager, and the final shared consultation between patient, GP and care manager. After each consultation, the care manager and/or the GP were asked to comment on it. The patients also agreed to participate in observations and interviews. Findings related to the patient perspective will be published elsewhere.

In both studies, observations of treatment practices included face-to-face encounters and consultations by phone or email. Observations focused on interactions/relations, treatment options, cross-sectorial cooperation, treatment decisions and GP-patient negotiation thereof.

All observations were initially jotted down and later extended into written field notes [25, 26]. To eliminate potential misunderstandings, observations were followed up by short ethnographic conversations with the GP, care manager or patient, and sometimes with all of them in individual informal conversations.

#### Interviews

For the multi-practice study, we developed separate interview guides for GPs and clinic staff for the semi-structured interviews. Interviews focused on experiences with and perspectives on existing treatment possibilities and limitations both in general practice and in collaboration with specialised psychiatric care. In the single-case study, we performed two semi-structured interviews with the GP and the care manager: one before initiating the DDCM and one follow-up interview to provide in-depth insight into their views on enablers and barriers for the implementation and function of the intervention. Furthermore, all GPs across the two studies were asked which kind of support and treatment options were needed to improve the treatment of patients with mental health problems and the role of collaborative care in this.

Interviews were conducted in the clinics during lunch break or after work. One practice nurse and one receptionist from the rural single-handed practice participated in a group interview. Two practice nurses from the other two practices participated in ethnographic conversations. Additional data was gathered and observations qualified by the GPs and the clinic staff through informal ethnographic conversations during the first author’s presence in the practices, usually between patient consultations and during lunch break [25]. Interviews were recorded and transcribed verbatim. Practice nurse and secretary perspectives are not addressed in this paper.

Interviews with care managers centred on their experiences with usual treatment and usual cooperation with general practice (not related to the DCCM), on their expectations to the DCCM and their actual experiences with the DCCM. Both care managers collaborated with several different DCCM-participating practices; this offered additional, although second-hand, data on GP attitudes towards the DCCM. The care managers also provided their views on DCCM organization and implementation in various GP settings. Thus, the perspectives of the care manager in the single-case study are not exclusively related to the single-case study. Letting the care manager draw on the experiences with the DCCM across various general practice settings helped broaden the insight into barriers and enablers related to the DCCM. The same broadening of insights was gained by including the perspectives of the second care manager. Interview guides for interviews with care managers and GPs are shown as Additional file 1.

#### Analytic procedures

The analysis was conducted in an iterative process to closely connect data collection and analysis [26, 27]. The thematic analysis began from the initial review of literature and the preparation of research questions, over data collection, followed by pattern identification, interpretation and the final writing of this paper. Each interview transcript was read and reread to perform an initial coding. Key phrases and themes were compared across transcripts. Field notes based on observations in clinical settings and at DCCM recruitment meetings were included to provide a contextual framework for understanding the barriers. This procedure identified emerging insights and dominating themes [27], which were discussed and condensed by the research team. Scope and design of data collection were discussed throughout the course of fieldwork by the first and last authors. The first author conducted the initial coding and identification of themes. Final identification of themes, analytical choices and the framing of the paper were discussed by the research team before the writing of the first draft and in several iterations hereafter. All authors have contributed substantially to the writing of the manuscript.

#### Ethical considerations

The study followed the Code of Ethics by the American Anthropological Association [28]. The study was approved by the Danish Data Protection Agency (file number: 2014–41-3207), and data collection and data handling were performed in accordance with their guidelines. According to Danish law, no ethical approval from the regional Committee on Health Research Ethics was not needed for this study as no biomedical intervention was performed.

In accordance with existing guidelines and research ethics, all GPs, staff, and care managers received written information about the study and gave verbal consent before initiation of the study. All participants were informed about the purpose of the study, anonymity, that participation was voluntary, and that participants could withdraw from the study at any time. In addition, informal meetings before initiation of study activities were held with the participating general practices to inform GPs and staff about the study and its implications. Written information about the study was posted in the waiting areas of the general practices.

The data material produced and analyzed as part of the study is not publicly available due to ethical considerations. We constantly strive to ensure the full anonymity of participants, and we respect the verbal agreements made with the participants. As they were not asked for permission to make entire raw data material publically available, we find it ethically inappropriate to make interview transcripts and observational study notes publicly available. Data material is available from the corresponding author on reasonable request.

## Results

Three main sub-themes emerged from the analysis across the single-case study and the multi-practice study: 1) DCCM enablers, 2) DCCM barriers and 3) Who needs collaborative care? The relevance of target groups.

### DCCM enablers

#### Access to “free” treatment and workload reduction

GPs in both studies were positive towards a new treatment model addressing the growing number of patients with mild/moderate depression and anxiety, as these patients are time-consuming:

“*It would in fact be a relief to many general practices, no doubt about it, if you could* [refer patients]” (GP, multi-practice study).

“*I could easily give up those conversations* [conversational therapy]*…because we are under quite a pressure staff-wise, so it* [treatment by a care manager] *would be a really nice relief. If I had lots of time I might not give it* [conversational therapy] *up*” (GP, multi-practice study).

Most GPs expressed frustration about limited referral options for these patients and long waiting lists for treatment by private practising psychologists. The care managers added that the DCCM meets a need in general practice for patients who are not sick enough for treatment in specialized psychiatric ambulatories. A care manager explained:

“*There are certainly not any patients* [included in the DCCM] *with severe depression, and then they have no chance of being offered treatment in a psychiatric ambulatory. Alternatively, the GP should treat…and to my knowledge most GPs have done… these patients with conversational therapy, and now they have handed them over to the project* [the DCCM] *for a while*” (care manager).

Improved access to treatment and “free” treatment were important enablers among GPs and a reason for the single-case GP to join the DCCM:

“*I joined the project to have an offer to patients who cannot afford a private psychologist and who are not sick enough for treatment at the psychiatric hospital ambulatories*” (GP, single-case study).

The DCCM also targeted another GP concern; the existing reimbursement structures for provision of conversational therapy to patients with mild/moderate anxiety and depression. Danish GPs receive reimbursement for conversational therapy only if the patient receives at least two consultations. If a patient does not return for a second conversational therapy consultation, the GP is not entitled to reimbursement for the time spent on the first consultation; this can have a negative impact on the motivation of some GPs:

“*I allocate extra time and then maybe the problem is solved or the patient doesn’t show up* [for the next appointment]*, so I don’t get the reimbursement for the extra time spent…so I think rather despondently that I won’t engage in that*” (GP, single-case study).

The DCCM does not only accommodate workload issues concerning the many patients with mild/moderate anxiety and depression; it also tends to encompass the frustrations related to reimbursement structures.

#### Quality of treatment

In addition to workload issues, some GPs expected the CC treatment to improve the quality of care because the DCCM allowed more time for systematic cognitive conversational therapy. In the single-case study, the GP reported the impression that the care manager in fact improved the quality of care:

“*Well, she* [care manager] *has more time, and I think she works more thoroughly than I do. I don’t do it as thoroughly*” (GP, single-case study).

In line with this, a GP in the multi-practice study reflected on the advantages of collaborative care:

“*It would be really nice to feel that they* [care managers] *are competent and hired directly to deal with this and to have allocated time slots, where this person* [care manager] *could offer half an hour... And this person* [care manager] *could reach some level of overview of our patients, and get some kind of knowledge about these patients and have a continuous relation to them…that would be fantastic, and it would take off some of the pressure*” (GP, multi-practice study).

Most GPs across our studies agreed that the interest and expertise in mental health treatment vary immensely among GPs and that improved referral options would benefit both the GPs and their patients:

“*When you open a referral option, most GPs who do not find it interesting to work with mental health treatment will hurry to refer* [their patients]. *That is great, because everybody would be happy… And not everyone* [every GP] *has the competencies required in this field, or an interest…*” (GP, multi-practice study).

The lack of interest among some GPs combined with the need for improved quality in this field and the experience/knowhow of care managers could thus be another enabling factor for collaborative care.

#### Training and supervision by psychiatrist

GPs at the DCCM information meetings showed interest in the possibility for supervision and/or training by specialized psychiatrists. They especially requested brush-up courses on psychoactive drugs and regular supervision in peer groups lead by a psychiatrist. In the multi-practice study, GPs also stressed the importance of collaborating with a psychiatrist for specialised advice on patients who do not respond to the usual treatment in general practice. The same was expressed by the GP in the single-case study, but eight months after the DCCM launch she had not yet been introduced to brush-up courses or supervision by a psychiatrist.

The GPs’ need for upgrading their psychiatric competencies might be an enabling factor of the DCCM if a psychiatrist facilitates training and supervision.

#### Care managers as implementers

The care managers proved to be translators of the overall idea of the DCCM. This task was not a described standardized element of the DCCM, which left the care managers to pragmatic maneuvering. Observations of GP-care manager interactions and interviews with the two care managers indicated that the care managers aimed to facilitate a smooth implementation of collaborative care, but they also acknowledged the challenge of entering general practice as outsiders:

“*…it is about how you enter general practice, you enter their territory, and you come as a guest. So you have to tread cautiously …*” (care manager).

Approaching general practice in pragmatic ways and fitting the DCCM to the individual GP’s preferences supported the implementation of the DCCM. The care managers had to adapt to different appointment systems, lack of office space, different therapeutic approaches and varying levels of engagement because they worked in many different clinics. The care managers also adjusted to the priorities of the GPs. For example, the GPs were to complete standard questionnaires with the patients, but the care managers took on this task when this did not happen although it deviated from DCCM “protocol” to get things done and avoid conflict:

“*We* [the care managers] *do take it* [the workload of GPs] *into consideration. Maybe it is overstated… I have defended this* [doing the GP’s task] *to myself by thinking that it is extremely important that we get started well and that we get to know each other, and that I don’t appear too rigid and too insisting*” (care manager).

The flexible and pragmatic approach taken by the care managers enabled implementation of the DCCM. The ability to juggle GP needs and adapt DCCM elements to different organisations made the care manager an asset in collaborative care.

### DCCM barriers

#### Organizational and practical barriers

The DCCM caused several logistic and organizational problems that (although simple) caused trouble in the implementation. First, the referral procedures were inadequately described:

“*It caused me trouble. The first few times I just referred* [the patient] *to the psychiatric hospital with a note saying that this person wanted to participate in collaborative care, and then they declined because referral requires a scheduled time for consultation with the care manager…and I don’t think I have been properly informed about that*” (GP, single-case study).

Second, there was lack of information about the practical procedures on how to obtain remuneration:

“*We have these reimbursement options which I have tried to apply, and then they are rejected, apparently because I am not registered in the care manager project. Well. Who do I register with? So I just had to contact them again and claim to be registered in the project and then they accepted…I have contacted the project manager twice about this, and he hasn’t replied…we need some follow-up on the information meeting*” (Single-case GP).

Third, the DCCM increased the GP workload as it required shared consultations with the care manager, coordination and booking of consultations, and setting up appointments for patients. It also proved difficult to organise a consultation room for the care manager:

“*Well, there is all the hassle; everything I have attended…information meetings, meetings* [with the care manager] *here and…yes, making sure the consultation room is ready. There is a lot I have to do extra…*” (GP, single-case study).

In line with this, a care manager stated:

“*They don’t have a spare room, so there is a lot of logistics and planning in it for us as care managers. My calendar, the patient’s calendar and then the consultation room, the* [psycho-education] *classes, conferences. It is a huge logistic work. And some patients go to school and some go to work, which you must also show consideration for*” (care manager).

In the follow-up interview, the GP in the single-case study concluded that she might not want to volunteer and invest the extra resources in a similar future collaborative care model.

The findings indicate that the DCCM was inadequately prepared; the project did not get off to a smooth start, and this challenged successful implementation and continuation of the intervention.

#### Cross-sectorial collaboration

An important aim of the DCCM was to improve the cooperation and communication between primary and secondary care through facilitation by care managers. Although most GPs in both studies embraced the idea of collaboration with specialised psychiatry, several GPs at the recruitment meeting expressed a priori scepticism towards handing over their patients to care managers. Furthermore, some were reluctant to attend training courses facilitated by care managers. This a priori scepticism towards the role of care managers in cross-sectorial collaborative care can be interpreted as a mental barrier to the DCCM and as a sign of a lack of interprofessional respect. In line with this, a GP in the multi-practice study expressed a general frustration towards existing visitation procedures where psychiatric nurses assess whether a patient referred by a GP is eligible for specialized psychiatric care:

“*It can be frustrating when you refer a patient who doesn’t respond to the treatment, and then it is a nurse who assesses the patient and rejects the patient without having a psychiatrist involved*” (GP, multi-practice study).

The quote could be read as lack of confidence in the expertise of psychiatric nurses, but it also expresses a frustration in the GP towards what seems to be gatekeeping by psychiatric nurses when the GP needs assistance by a psychiatrist. This frustration was shared by most GPs in both the single-case study and the multi-practice study. Despite an a priori scepticism in some GPs, the GP in the single-case study expressed that her clinical cooperation with the care manager in the DCCM ran smoothly and that the care manager seemed to be competent. The care manager herself experienced a positive attitude towards her in most of the DCCM practices she collaborated with, and she found that the GPs actually welcomed interdisciplinary exchange:

“*What I hear, at least from three of the GPs that I talked to, is simply a need for a professional back-and-forth; the dialogue, ‘I see the case* [the patient] *like this and this: How do you see it?*” (care manager).

Despite the willingness in the GP to provide a professional back-and-forth, the DCCM set-up did not facilitate a closer cooperation according to the findings in the single-case study. Except for brief ad hoc exchanges of treatment plans and coordination of shared consultations, both the GP and the care manager considered it more as a *transfer* of the patient. In that sense, as also reported by the single-case GP, the collaborative care project facilitated only a shallow relationship across sectors and disciplines.

The GP stated:

“*I don’t cooperate a lot with the care manager in the sense that we exchange experiences and stuff … I get to know the care manager a bit through the initial and last consultations. But proper exchange of knowledge and experience on how to handle these patients or sharing her tricks with me…that doesn’t really happen*” (GP, single-case study).

Both care managers agreed. One care manager concluded:

“*The way that our shared consultations worked meant that they actually had the characteristics of a transfer* [of the patient] *with the GP telling the patient, in my presence, that ‘I have told this and this* [to the care manager] *about your situation’*” (care manager).

The other care manager said:

“*…and my conclusion right now is that it is hard for me to see, in relation to these patients, why I should claim a need for a shared consultation*” (care manager).

The care managers and the GP agreed that written communication would have been sufficient to exchange the necessary information and that treatment of patients by care managers could have been conducted in a psychiatric outpatient ambulatory, but the collaborative care part would then have vanished. One care manager explained:

“*They* [the patients] *might as well get treatment in the ambulatory, but then there would be no collaboration with general practitioners*” (care manager).

The GP in the single-case study acknowledged that increased cooperation across sectors was time consuming, and she was unsure if she would be willing to invest more time in it.

In addition to organisational and logistical issues, several other important barriers to the DCCM were identified: the unclear character and purpose of the cross-disciplinary cooperation, a priori scepticism in many GPs towards care manager-facilitated training, and limited actual exchange of knowledge and experience.

#### Sustainability

The single-case study revealed a number of organisational, practical, logistic and resource-related barriers to the DCCM when meeting the realities in general practice. The care managers were intended to provide treatment of patients in up to 10 general practices [23]. This set-up challenged both coordination and logistics, and the care managers experienced a considerable waste of time when commuting between different general practices. Due to these barriers and the lack of capacity in specialized psychiatric care, the care managers doubted that the model would be sustainable beyond the project period:

“*There wouldn’t be enough qualified nurses if you deploy this model everywhere. So I can’t imagine how this model should be implemented permanently in its current form. I really don’t*” (care manager).

The GPs in the multi-practice study appreciated the potential of the DCCM to free time, but some also feared that it might “steal time” due to planning and coordination with the care manager. The care managers found it time-consuming to get the DCCM model started and running (also after the run-in period).

The logistical problems of finding a vacant consultation room for the care manager were also mentioned as a potential barrier to the sustainability of the proposed DCCM by GPs in the multi-practice study:

“*It* [treatment by care manager] *should not be done here. Then they* [the care managers*] would have to be here all the time. Then they* [the care managers] *would need their own consultation room, or we should receive some kind of reimbursement, or we should rent out* [the consultation room], *and then we would have the trouble with administrating their booking of consultations and things like that. So… otherwise, I would be the one paying for the patients’ free treatment, and then we wouldn’t do it. It* [collaborative care] *has to be solved* [done] *somewhere else, I think…then it would be really great, but yes…they should create an ambulatory for it because it would be swarmed with patients*” (GP, multi-practice study).the multi-practice study

One important barrier thus seems to be that the DCCM involved extra (rather than reduced) workload in general practice without compensating the GPs for this additional work.

Several barriers to the DCCM were experienced by the single-case GP and perceived by the GPs in the multi-practice study. Although the care managers played a central role in the treatment and collaboration related to the DCCM, none of the GPs in the multi-practice study requested elaborated cooperation with a care manager from specialised psychiatry for patients with mild/moderate anxiety and depression. The GPs seemed to expect benefits from collaborative care in terms of improved access to high-quality treatment rather than increased collaboration with external partners, including a care manager situated in the clinic. Consequently, the support gained by the DDCM was not considered to sufficiently justify the time spent.

#### Who needs collaborative care? The relevance of target group

DCCM enablers and barriers were partly related to the way that the GPs perceived mental healthcare for different patient groups, and how the GPs tended to divide these into categories according to the severity of the patients’ mental illness and their treatments needs.

When asked which of patients with mental health problems they found the most challenging to treat, the GPs (across both studies) referred to specific diagnoses, and they categorised the patients into two main groups based on the severity of conditions: 1) a large group of patients with mild/moderate mental health problems and 2) a small group of patients with severe mental health problems (e.g. severe depression, schizophrenia and other psychotic conditions) and higher risk of somatic comorbidity and complex health problems:

“*There are not as many patients with schizophrenia as patients with anxiety and depression so it is a matter of prioritising resources. The schizophrenic patients are left more to themselves; they are a more vulnerable group who rarely consult their GP. Patients with anxiety and depression consult their GP regularly. And often they have relatives who support them, whereas the schizophrenic patients are more socially isolated*” (GP, multi-practice study).

The GPs considered treatment of mild/moderate mental health conditions to be a central GP task, but the number of patients is increasing, and the GPs do not have the capacity to handle the volume of patients. The GPs emphasized that the complexity of the mental/somatic/social problems in patients with severe mental health conditions makes them an especially vulnerable group; they are the most challenging to care for in general practice and have the highest need for improved treatment.

“*Where are the schizophrenic patients now? The number of hospital beds have been cut back, they are discharged, and they disappear into nothing, and we are not in control of them…it happens too often*” (GP, multi-practice study).

When asked about the potential of a collaborative care model that integrates psychiatric and general practice treatment, the GPs agreed that the patients with severe mental health problems and somatic comorbidity have the highest need for cross-disciplinary and collaborative treatment. The complexity of health conditions in these patients calls for new ways to ensure integrated mental healthcare that meets their somatic, psychiatric and social needs, whereas the GPs did not see a need for enhanced collaboration with specialised psychiatry on patients with mild/moderate mental problems. For this patient group, they requested a fast track to high-quality treatment in cases when usual care fails. This discordance between the target group of the DCCM and the actual and perceived needs of collaborative care suggests a major motivational barrier for successful implementation of the DCCM.

## Discussion

### Main findings

We did a purposeful sampling for the multi-practice study in order to include potential variations in GP perceptions and their need for collaborative care. However, we found very limited variation. Therefore, the analysis centred on the thematic trends across the two studies. The introduction of the DCCM for mild/moderate anxiety and depression was promoted by a need in the GPs for better access to free high-quality treatment and pragmatic manoeuvrings by the care managers. Major barriers to the implementation of the DCCM, as experienced by the single-case GP and the care managers, were organisational deficiencies concerning patient registration and reimbursement procedures, limited genuine collaboration and knowledge sharing, much GP time spent on practical issues and little availability of consultation rooms. Another barrier perceived by the GPs in the multi-practice study and, to some extent, by the single-case GP was the lack of confidence among GPs in the importance of investing time and resources for enhanced cooperation across sectors and disciplines on this patient group; most participants questioned the sustainability of the DCCM.

As an overall framework for treatment of patients with mental health problems, the DCCM fits well with the dominant discourse in modern western healthcare policy, which focuses on coherent, cross-disciplinary and cross-sectorial healthcare set-ups. However, it has been argued elsewhere that the societal focus on cooperation across traditional sector boundaries cannot be expected to be mirrored in everyday life [29, 30]. This may explain why carefully developed collaborative care models may work under experimental conditions but not in real-life situations, as we also found in this study. Such models collide with daily life in general practice, where GPs must make meaningful choices with limited resources; all activities are subject to standardised fee-for-service agreements, and all choices are influenced by competing tasks, different patient needs and individual preferences among GPs and patients.

If the GPs consider improved referral options and not collaboration with a care manager to be the most important for this patient group, the DCCM should reflect this. The logistic and financial constraints also need to be addressed. The GPs were not per se against the concept of collaboration, but they wanted cooperation and communication to fit with their practice life, and they were concerned about potential side effects in terms of increased workload. In particular, the introduction of an external care manager in general practice revealed logistic, practical and financial barriers.

The practical daily life of general practice should be taken as a starting point when a revised DCCM is developed and implemented. Marshall states that “improvement initiatives are sometimes planned on the hard high ground, but they are put into effect in the swampy lowlands” [31]. Therefore, central stakeholders, such as GPs and patients, need to be deeply involved in the development of new collaborative models to ensure that they are designed to meet the existing organisational, cultural and structural realities [32]. Our findings also indicate the importance of developing a DCCM in a systematic way, including pilot testing as described by the Medical Research Council in the guidance on the development of complex interventions, in order to adjust new intervention prototypes to the clinical reality of general practice before presenting a new model [33, 34].

The two-armed research design that combines the DCCM single-case study with a multi-practice study allows the discrepancies between model and reality to emerge. We wanted to take a bottom-up approach to the need for collaborative care in contrast to the top-down developed DCCM in order to discuss and problematise the introduction of new models without involving all relevant stakeholders.

### Comparison with other studies

Our findings are consistent with other studies on the implementation of collaborative care in both the UK, the US and the Netherlands, which show that it can be challenging to implement research-designed collaborative care in real-life general practice settings [9, 12, 13, 16, 35, 36]. Other studies have found that collaborative care can be implemented if financial barriers are reduced, effective training and facilitation are provided, a common mental health model is developed, and new care processes are introduced [7]. The most important factors found in other studies using the Normalisation Process Theory as the analytical frame were shared understanding of mental illness between GPs and care managers and agreement on the division of tasks [4, 12]. Our findings support the crucial role of developing a common understanding of mental health conditions and their treatment as well as targeting logistic and financial barriers [37]. Adding to this, our study provides insight into the GPs’ distinction between mild/moderate and severe mental illness. Treatment of mild/moderate conditions can be optimised within general practice and through more referral options to psychological therapy, whereas collaborative care is perceived to be more suitable for severe cases.

### Strengths and limitations

The present study has limitations with regard to generalisability. The general practices in the study may not cover all types of practices, and the included GPs were known to be among the most progressive and active compared to other general practice clinics. Therefore, we would expect them to have a more positive attitude to new models of care delivery, such as a DCCM. Consequently, our findings may report a more positive attitude to the DCCM than would have been found in the general population of GPs.

Financial constraints allowed us to include only one GP clinic participating in the DCCM. Thus, the findings reflect the experiences from only one collaborative care setting and may not be representative of collaborative care experiences in other general practices. However, a broader perspective on the function of the model was ensured through the inclusion of two care managers as both of these interacted with several GPs across different general practice settings and with numerous patients during the first six months of the intervention. Moreover, the model was observed throughout the entire process (including information meetings, initial meetings between care manager and GP and the entire course of treatment) and through a combination of before and after interviews. This thorough exploration added strengths and depth to the study by providing broad and multi-facetted perspectives on the functioning of the model. Furthermore, many of the experiences from the single-case study accorded with the findings from the multi-practice study.

## Conclusion

The increasing number of patients with mild/moderate anxiety and depression in general practice supports the implementation of collaborative care in mental health treatment. Additionally, the workload-reducing potential of care manager-led treatment did in fact constitute an actual and perceived enabling factor of the DCCM. Nevertheless, there are a number of experienced and suggested barriers to the sustainability of the DCCM in its current shape. Firstly, the GPs in this study did not request increased cooperation with specialised psychiatric care (represented by care managers) for patients with less severe mental conditions although they see a growing need for improved referral options for this patient group. Secondly, our single-case study suggests a number of practical and logistic barriers to the DCCM. Thus, the prospected barriers and concerns of GPs in the multi-practice study who had not experienced the implementation of the DCCM supplement the findings from the single-case study.

GPs and patients should be key in the development of future collaborative care models. The pragmatic reality of general practice, cross-sectorial differences in the perceptions of target groups, (lack of) needs for more cooperation, time constraints and identification of practical and logistical barriers should be considered to ensure successful implementation for all involved parties. Therefore, we argue that there is a need to ask: Who needs collaborative care?

## Additional file

Additional file 1: Interview guides. (PDF 126 kb)

## Abbreviations

DCCM: Danish collaborative care model
GP: General practitioner
