# Supplementary material for: Who needs collaborative care treatment? A qualitative study exploring attitudes towards and experiences with mental healthcare among general practitioners and care managers
Source: BMC Fam Pract. 2018 May 30;19:78. doi: 10.1186/s12875-018-0764-z (PMC5977752; doi:10.1186/s12875-018-0764-z)
Supplement: Supplementary file 1 — Interview guides. (PDF 126 kb) [file 12875_2018_764_MOESM1_ESM.pdf]

Interview guide for GP (multi-practice study)

Understanding of patient categories

- When we talk about patients with mental illness, which patients do you then think about?
- Which of these patients are most difficult to handle in general practice? Why?
- How do you experience that this is expressed in the patients?
- When we talk about patients with mental illness and coexisting physical disease, which patients do you then think about?
- How do you experience diagnosing and treating several coexisting diseases?
- How do you prioritize between the different diseases in the consultation? Why?

Treatment – current challenges and successes

- Which challenges do you see as the biggest and most important ones for patients with mental illness?
- What goes well? What are the successes? Why does it go well, do you think?
- Could you try to describe the course of events for such patient?

Proper/good treatment & negotiation thereof

- What should good/proper treatment consist of for these patients? How should it be carried out?
- What do you need (as a GP) to improve the treatment of these patients?
- What could obstruct this? Who should be responsible?
- Do you and the patients agree on what good treatment is? If not, what do you then do?
- What do you think the patient needs? Is it possible to give this to the patient today?

Cross-sectorial collaboration (perspectives and perceptions of collaborative care)

- Is treatment of mental disorders a core competence in general practice or?
- Which collaborative areas are important to provide this patient group with proper treatment?
- Which collaborative areas do you miss/should be strengthened? Why?
- What characterizes good cross-sectorial collaboration? What is required? What can obstruct a successful collaboration?
- Do you agree across sectors on how to best treat patients with mental illness?
- Which components are decisive for making a future collaborative care model work? (The DCCM in the single-case study is presented to the GP in relation to this question)
- Which barriers do you see for a successful implementation of such model?
- How is your approach to potential collaboration with care managers from psychiatry? Why?

Interview guide for GP (single-case study) prior to collaborative care

Understanding of patient categories

- When we talk about patients mental health problems, which patients do then come to your mind?
- Which of these patients are most difficult to handle in general practice? Why?
- How do you experience that this is expressed in the patients?

Treatment – current challenges and successes

- Which challenges do you see as the biggest and most important ones for patients with mild to moderate anxiety/depression compared to patients with other mental health problems?
- How are these expressed in daily clinical practice? In the treatment?
- What goes well? What are the successes? Why does it go well, do you think?
- Could you try to describe the course of events for such patient?

Proper/good treatment & negotiation thereof

- What should proper/good treatment consist of for these patients? How should it be carried out?
- Which treatment components should be included in proper/good treatment of this patient group?
- What do you need (as a GP) to improve the treatment of these patients?
- What could obstruct this? (Practical, organisational, financial obstacles?)
- What do you think the patient needs? Is it possible to give this to the patient today?
- Do you and the patients agree on what good treatment is? If not, what do you then do?

Cross-sectorial collaboration (expectations to collaborative care)

- Within which framework do you believe that the treatment of these patients belongs? Is treatment of mental disorders a core competence in general practice or?
- Which collaborative areas are important to provide this patient group with proper treatment?
- Which collaborative areas are missing/should be strengthened? Why?
- What characterizes good cross-sectorial collaboration? What is required? What can obstruct a successful collaboration?
- Do you agree across sectors on how to best treat patients?
- Why did you sign up as a participant in the collaborative care project? And what are your expectations?
- Which components are decisive for making a future collaborative care model work?
- Which barriers do you see for a successful implementation of such model?
- How is your approach to the collaboration with care managers from psychiatry? Why?

Interview guide for GP (follow-up interview) on experiences with collaborative care

Recruitment

- How many of your patients have completed the collaborative care programme? How many are currently going through it?
- Can you still recruit patients for the project?
- How was it to identify patients for the project?
- Was it easy to define who belonged in the project? Why/why not?
- How did the patients react to this offer?
- Did any of the invited patients decline to participate? How many? Why?
- Was there any difference in the participation between women and men?
- Were there equally many patients with anxiety and with depression? Why do you think that is?
- Could you try to describe what happens from the time when you are to find a patient and until the programme has been completed?

Organisational issues

- How did the collaborative care project fit in with your daily organisation of the work?
- At which areas has the project had an influence?
- Did the project intervene in any way, i.e. did it require changes in your way of doing things?
- What did the receptionist think about being part of the project and the collaboration with the care manager?
- Did the project give you or your receptionist any extra tasks? How?
- How did you solve it?
- What does it mean to you that the conversations take place in your general practice? Why?

Treatment

- How did it work to leave the treatment of your patients to a care manager?
- What do you think about getting the patients back for usual treatment with you?
- How do you assess the effect of the treatment programme that the patients go through?
- Which treatment elements are described in particularly positive terms – by the patients? by the care manager? by you? by your colleagues?

Cross-sectorial collaboration

- What is your experience of the collaboration with the care manager?
- Where does it work well? Why?
- Where could it (perhaps) work better? How?
- How did you follow the patients during the project?
- Did you have a systematic approach to reading the care manager's correspondence letter? Why/why not?
- Were the correspondence letters sufficient, or did you need to supplement with other types of communication?
- Did you meet face-to-face? How did this fit in with your other work?
- Did you need to have joint meetings? In which cases?
- Did you need to request professional back-and-forth with a psychiatrist?
- Did you receive any kind of instruction/training in relation to the project?
- What do you think about that?

Practical issues

- Were there any practical challenges related to being part of the project?
- How was this expressed?
- How was it solved?

Full implementation

- Did the project meet your expectations? How? Why not?
- Do you think that the project suits well with a practice type such as yours?
- What needs to be changed to make it work?
- What worked well?
- Which opportunities do you see for a future collaborative care model?

Physical comorbidity

- Did any of the patients who completed the collaborative care project have (or were at risk of developing) physical disease?
- Which challenges/possibilities do you see in terms of treating these?

Perceptions of 'proper' treatment

- From your perspective, what is good and proper treatment of patients with mental illness?
- Do you see the same treatment focus in the care manager as in a representative for psychiatry?
- Which discussions did you have concerning the treatment of the patients? Disagreements?

Interview guide for care manager (single case study) prior to collaborative care

About you

- Could you start with telling me a little about yourself and your work here?
- What was your field of work before collaborative care?
- How did you become part of the project?

Patient category

- When we talk about depression and anxiety, which conditions do you then think about?
- How do these differ from the patients who are usually referred from the GP?
- How do you experience that depression and anxiety show in the patients?

Treatment

- How do you usually handle patients with depression and anxiety?
- Do you usually treat patients with mild to moderate anxiety and depression within the scope of your profession?
- What is good and proper treatment of these patients?
- Do you use any specific method?
- How is a typical treatment course?
- Which treatment elements do you regard as the most important?

Physical comorbidity

- How do you experience the patients' physical health?
- Is it often physical comorbidity?
- Is this something you address or collaborate with the GP about?
- Which challenges do you see that it could give in relation to the treatment?

Cross-sectorial collaboration

- How do you perceive the collaboration with the patients' own GP (before collaborative care)?
- What are your expectations to the collaborative care project?
- Which pros and cons do you see about the model?
- How do you imagine that the model could influence general practice?
- How do you imagine that the model could influence psychiatry?
- What do you think could ensure a good collaboration?
- What do you think could obstruct a good collaboration?

- How do you think that the model will be received in general practice?
- What do you find important to ensure that the two sectors receive the model well?
- Do you think that GPs, psychiatrists and care managers will have a common understanding of the task relating to the patients? Both concerning patient group and collaboration?
- What do you think it would take for the parties to reach a common understanding of the task relating to this patient group?
- Do you think that GPs, psychiatrists and care managers have a common understanding of how to collaborate on the model?
- What do think is the care manager’s most important function in the collaborative care model?

Interview guide for care manager (follow-up interview) on experiences with collaborative care (single-case study)

Recruitment

- How many patients have completed the collaborative care programme? How many are currently going through it?
- Can you still recruit enough patients for the project?
- Are the referred patients in the target group? Why/why not?
- Have the patients expressed any expectations to the programme?
- Did any of the invited patients decline participation after the first interview? How many? Why?
- Was there any difference in the participation between women and men?
- Why do you think that is?
- Are there equally many patients with anxiety and with depression? Why do you think that is?
- How did you experience the recruitment of participating GPs? Why?

Organisational issues

- Was the project easy to get started in clinical practice? How?
- How did the collaborative care project fit in with everyday life in general practice? Describe!
- Are there any differences between which [types of] practices the project suited best? Size, organisation or similar?
- Do you find that general practice must change their procedures to accommodate the project?
- Which collaborative areas are most decisive for your work? GP, receptionist?
- How is this expressed?
- What do you think that it means to the patients that the conversations take place at their own GP? Why?

Treatment

- How did it work to take over the treatment of patients?
- How do you think that the handing over of patients to own GP has worked?
- Should other ways of handing over be considered?
- How do you assess the effect of the course of treatment that the patients go through?
- Which treatment elements work especially well?
- What were the reactions from patients, GPs and your colleagues?

Cross-sectorial collaboration

- How did you experience the collaboration with the GPs?
- Where did it work well? Why?
- Where could it be improved? How?
- How have the GPs been involved?
- Was this involvement sufficient?
- Did you take a systematic approach to informing the GPs during the project? Why/why not?
- Did the project hold any scheduled professional back-and-forth (e.g. joint meetings or similar)?
- Did you meet face-to-face? How did this fit in with your other work?
- Was there any need for joint meetings? In which connections?
- How was the psychiatrist involved?
- Did the project include any kind of teaching directed towards GPs?

Practical issues

- Which practical challenges were there in connection with the project?
- How were these expressed? And how was it solved?
- Is there something that you think should be addressed if the project is to be broadly implemented?

Full implementation

- Did the project meet your expectations? How/why not?
- Do you think that the project fits well in general practice?
- What needs to be changed for it to work?
- What worked well?
- Which opportunities do you see for a future collaborative care model?
- How did the collaboration between you care managers and the project management work?

Physical comorbidity

- Did any of the patients who completed a treatment programme as part of the project have (or were at risk of developing) physical disease?
- Which challenges/opportunities do you see in terms of treating these?
- Could the care, supervision, control of physical diseases or prevention of physical diseases be thought into a collaborative care model? How? Why not?

Interview guide for care manager (other municipality)

About you

- Could you start with telling me a little about yourself and your work here?
- What was your field of work before collaborative care?
- How did you become part of the project?

The patient category

- When we talk about depression and anxiety, which conditions do you then think about?
- How do you experience that depression and anxiety show in the patients?
- How do the patients in the project differ from the patients who are usually referred from the GP?

Treatment

- What is good and proper treatment of patients with anxiety and depression?
- Do you use any specific method?
- What does a typical treatment course look like?
- Which treatment components do you consider as the most important?
- Is there any difference between your usual approach and the treatment given to patients in collaborative care?

Physical comorbidity

- How do you experience the patients' physical health?
- Is there something specific that you address or collaborate with the GP about?
- Which challenges do you see that physical comorbidity could give in relation to the treatment?

Cross-sectorial collaboration

- How do you perceive the collaboration with the patients' own GP? (before/outside collaborative care)
- What were your expectations to the collaborative care project before you started?
- Which pros and cons do you see about the model?
- How do you expect the model to influence general practice?
- How do you expect the model to influence psychiatry?
- What do you think would ensure a good collaboration?
- What do you think could hinder a good collaboration?
- How do you experience that the model has been received in general practice?

- How has it been received in psychiatry - in general?
- What do you find important to ensure that the two sectors receive the model well?
- Do you think that GPs, psychiatrists and care managers have a common understanding of the task in relation to the patients? Both concerning patient group and collaboration?
- Do you think that GPs, psychiatrists and care managers have common understanding of how to collaborate on the model?
- What do you see as the care manager's most important function in the collaborative care model?
- Based on your experience so far, do see any practical or organizational barriers among GPs that could hinder rolling out of the model?
- Did you experience any practical or organizational challenges regarding the implementation of the model in practice?
- How do you think that the work with patients finished in collaborative care should continue? Will treatment still be based on collaborative care, or should the patients be finished by you?
